# LGBTQ+ Cultural Sensitivity Training for Mental Health Professionals in the USA

**DOI:** 10.1007/s10508-025-03132-3

**Published:** 2025-03-25

**Authors:** Juan C. Jauregui, Gary W. Harper

**Affiliations:** 1 Department of Social Welfare, UCLA Luskin School of Public Affairs, 3250 Public Affairs Building, Box 951656, Los Angeles, CA 90095 USA; 2 Department of Health Behavior and Health Education, University of Michigan School of Public Health, Ann Arbor, MI USA

## Introduction

Lesbian, gay, bisexual, transgender, and queer (LGBTQ+) cultural sensitivity in mental health care delivery within the USA is needed and can significantly impact the health and well-being of LGBTQ+ individuals (Mayer et al., 2008; Sekoni et al., 2017). Research shows that the LGBTQ+ community faces significant mental health disparities, such as increased risk for depression, anxiety, post-traumatic stress, and substance use disorders compared to their cisgender–heterosexual counterparts (Ross et al., 2018; Wittgens et al., 2022). Given the outcome of the 2024 US presidential election and the wave of executive orders with anti-LGBTQ+ implications signed the first day in office, there is a potential for increased mental health challenges in the next four years as was revealed by data from a national probability sample of sexual minority adults that demonstrated evidence of worsening mental health and social well-being in the 17 months after Trump’s last term in office for Black and Latinx sexual minority people (Krueger et al., 2021).

Data from two large national probability sample studies—the Generations Study and the TransPop Study—also suggest that LGBTQ+ people are at higher risk of suicide (Meyer et al., 2021). Disaggregated data from these studies show differences between subpopulations of LGBTQ+ people, with transgender people reporting the highest rate of suicide attempts (42%), followed by LBQ cis women (31.6%) and GBQ cis men (21.5%) (Meyer et al., 2021). In comparison, data from the US National Epidemiologic Study of Addictions and Related Conditions III (NESARC-III) find that the estimated prevalence of suicide attempts in the general US population is 5.2% (Oquendo et al., 2024). Due to these and other major health disparities, the National Institute of Health officially designated sexual and gender minorities as a health disparity population in 2016 (Pérez-Stable, 2016).

The increased psychological distress experienced by people who are LGBTQ+ has been partly demonstrated to be a result of societal stigma and discrimination (Meyer & Frost, 2013; Safren & Heimberg, 1999). Structural forms of stigma are the societal-level conditions, cultural practices, and institutional policies that bound the well-being of the stigmatized and have been described as an underrecognized mechanism producing health disparities for LGBTQ+ populations (Hatzenbuehler, 2016). The minority stress model is a framework that explains how experiences of prejudice, stigma, and discrimination on the basis of one or more marginalized social identities negatively impacts the mental health of minority communities (Meyer & Frost, 2013). The effects of minority stress can be further exacerbated for LGBTQ+ people who are also racial minorities, low-income, and/or undocumented due to experiences of intersectional stigma and discrimination (Balsam et al., 2011; DeSon & Andover, 2024).

Minority stress may explain part of the variance observed in poor mental health among LGBTQ+ populations, but additional non-LGBTQ+-specific factors must also be considered. General stressors may include academic difficulties, problems with romantic relationships, and financial difficulties. Multiple domains of stress (gay-specific and general) have been found to have a multiplicative effect that significantly impacts negative affect among gay men (Petruzzella et al., 2020). Recent research has further nuanced our understanding of minority stress by examining the unique and interrelated influences of gender minority stressors among transgender and gender diverse individuals. Cao et al. (2024) highlight how internalized transphobia, preoccupation with gender dysphoria, and gender-related victimization were particularly salient in shaping psychological distress. Additional research suggests adding rejection sensitivity to the characterization of LGBTQ+ mental health could complement minority stress theory and the psychological mediation framework (Feinstein, 2020); however, a focus on rejection sensitivity for LGBTQ+ populations has been critiqued as it places an emphasis on the individual rather than the social conditions that lead to adverse mental health outcomes (Meyer, 2020). Changing the social conditions in which stigma is experienced within mental healthcare settings is one approach that could promote LGBTQ+ mental well-being. Actions toward LGBTQ+ culturally sensitive healthcare could include ensuring that clinical intake forms are inclusive of sexual and gender diversity, educating providers on LGBTQ+ issues, or increasing access to LGBTQ+ -specific support groups. Unfortunately, all of these actions may be in jeopardy with the new federal administration.

Evidence suggests that LGBTQ+ cultural sensitivity trainings could improve clinical delivery to LGBTQ+ clients. Studies from European countries report that such trainings have been effective in increasing knowledge, awareness, and confidence in working with LGBTQ+ people among healthcare professionals (Donisi et al., 2020), and 63% of providers reported a desire to have this training included as part of their mandatory professional development (Burgwal et al., 2021). An evaluation on a LGBTQ+ cultural humility training found that providers made significant changes in individual- and clinic-level practices with LGBTQ+ people post-training, indicating the promise of this approach for translating knowledge into the improvement of clinical delivery for LGBTQ+ people (Jadwin-Cakmak et al., 2020). Such trainings typically introduce providers to skills such as self-reflectivity and openness to other cultures that would also be beneficial when working with diverse populations. Focusing on provider capacity building through LGBTQ+ culturally sensitivity trainings is key to transforming healthcare environments to help promote positive healthcare experiences for LGBTQ+ people while simultaneously increasing access to affirming mental health providers and services.

## LGBTQ+ Experiences in Mental Healthcare

LGBTQ+ people have a longstanding history of negative experiences with the mental health profession (Moleiro & Pinto, 2015). While contemporary practice has seen a rise in affirmative therapies that do not pathologize LGBQ identities (O'Shaughnessy & Speir, 2018), this is not universal across the USA. The situation is particularly stark for transgender individuals, who continue to experience growing restrictions on access to gender-affirming therapy and decreases in anti-discrimination protections (Warling & Keuroghlian, 2022).

A current source of tension between LGBTQ+ people and the mental health profession is the practice of sexual orientation and gender identity change efforts (SOGICE), often referred to as conversion or reparative “therapy,” which are pseudoscientific practices that attempt to change an individual’s sexual orientation, gender identity, or gender expression using professionally discredited practices that can cause great harm, especially among youth (SAMHSA, 2023). All US professional associations of mental health providers (e.g., American Psychological Association, American Psychiatric Association, American Counseling Association, and National Association of Social Workers) and major health-related professional associations (e.g., American Medical Association, American Academy of Pediatrics, and Society for Adolescent Health and Medicine) have policy and/or position statements opposing the use of conversion therapy. Estimates using national datasets (Youth Risk Behavioral Surveillance System, Behavioral Risk Factor Surveillance System, US Transgender Survey, US Census, and the Generations Study) show that 16,000 LGBT youth aged 13–17 will receive conversion therapy by a licensed professional before they turn 18 (Mallory et al., 2019).

Presently, fewer than half of the US have taken action to protect vulnerable LGBTQ+ youth by banning state-licensed mental health professionals from practicing SOGICE with minors (Mallory et al., 2019; Movement Advancement Project, 2025). A recent report by The Trevor Project (2023) identified more than 1320 current “conversion therapy” practitioners across 48 states and the District of Columbia, including more than 600 practitioners who hold active professional license, and more than 70 additional identified practitioners are interns or are in training for full licensure. Given the negativity that surrounds terms such as “conversion therapy” and “reparative therapy,” groups of mental health providers who still practice and support SOGICE have “re-branded” their practices. The Alliance for Therapeutic Choice and Scientific Integrity (n.d.), and its accompanying “scholarly journal,” *The Journal of Human Sexuality*, supports SOGICE with adults and the practitioners associated with them have renamed SOGICE “Sexual Attraction Fluidity Exploration in Therapy (SAFE-T).” Another group, the “Reintegrative Therapy Association” (n.d.) promotes the use of Reintegrative Therapy® which they state, is not designed to change a person’s sexual orientation but claim that changes in sexual orientation occur as a by-product of their “evidence-based” approach to treating trauma, and not as the goal of their therapy.

There is considerable debate regarding bans on the practice of conversion therapy and what legally constitutes the practice. The complexity of this issue is evident in ongoing legal challenges, such as the case in Michigan, where a lawsuit has been filed by religiously affiliated clinicians who argue that the state’s ban on conversion therapy infringes upon their freedom of speech and the right to offer counseling to youth in need (Michigan Department of Attorney General, 2024). A federal judge recently denied this group’s request for a preliminary injunction to block the enforcement of the conversion therapy ban while their lawsuit proceeds in Court, writing that the plaintiffs are unlikely to succeed on the merits of their case (Lobo, 2025). Michigan’s legislation banning conversion therapy specifically delineates this practice as, “any practice by a mental health professional that seeks to change an individual’s sexual orientation or gender identity” and explicitly does not include:

counseling, that provides assistance to an individual undergoing a gender transition, counseling that provides acceptance, support, or understanding of an individual or facilitates an individual’s coping, social support, or identity exploration and development, including sexual orientation-neutral intervention to prevent or address unlawful conduct or unsafe sexual practices, as long as the counseling does not seek to change an individual’s sexual orientation or gender identity… (Michigan Public Act 118, 2023).

This distinction is crucial as it highlights that psychotherapy designed to explore a young person’s gender identity or sexual orientation, while remaining neutral and promoting individual autonomy does not fall under the category of conversion therapy. A clinician working with a 14-year-old who expresses confusion about their gender identity, for example, could explore that confusion in a supportive, non-directive way without being considered to engage in conversion therapy. Sinai and Sim (2024) and others describe a growing concern among clinicians’ anxiety about being charged under criminal codes that violate conversion therapy bans as some therapeutic approaches may be misconstrued as conversion therapy. It is important that what is considered “conversion therapy” should be understood within the context of intentionality and harm: If the aim is to coerce a particular identity or suppress authentic self-expression, this is more likely constituting conversion therapy. On the other hand, a clinician who works with a young person to explore their identity, supporting them as they process their feelings without pushing an agenda, would not be engaging in harmful practice. To better understand these nuances and therapeutic approaches, clinicians could benefit from explicit training that clarifies how to offer culturally sensitive care that respects autonomy, supports identity exploration, and remains neutral to avoid pitfalls of coercion or ideological imposition.

Clinicians who are not sensitized to LGBTQ+ issues can cause harm in the form of microaggressions and invalidation of LGBTQ+ lived realities (Israel et al., 2008; McCullough et al., 2017; Spengler et al., 2016). Microaggressions can include refusing to use the correct gender pronouns for a client, referring a client who is a transman to a women’s support group, or assuming a client is talking about someone of the opposite sex when describing a romantic relationship (McCullough et al., 2017; Spengler et al., 2016). Negative experiences with practitioners serve as a barrier to healthcare for LGBTQ+ individuals and can lead to premature termination of the therapeutic relationship (Israel et al., 2008; Romanelli & Hudson, 2017). The therapeutic alliance also becomes strained when LGBTQ+ community members need to educate providers who are unfamiliar with how to navigate issues of sexual orientation or gender identity (McCullough et al., 2017). Moreover, for LGBTQ+ adolescents and young adults who are in earlier stages of their identity development, experiencing microaggressions from a mental health clinician can be particularly damaging as they are forming their LGBTQ+ identity and may result in feelings of rejection and internalized self-stigma (Spengler et al., 2016).

LGBTQ+ people have described helpful therapeutic interactions as those with therapists who appear to be knowledgeable and affirming when navigating clients’ sexual orientation and/or gender identity (Israel et al., 2008). While LGBTQ+ clients describe some general factors as helpful (e.g., warmth and trustworthiness of a provider), LGBTQ+ -specific factors include respecting a client’s name and pronouns, respecting their choice of who to come out to, and avoiding the assumption that a client’s LGBTQ+ identity is at the root of their presenting problems (Israel et al., 2008; McCullough et al., 2017). These positive experiences with healthcare providers have been associated with an increase in quality of life, coping skills, and increased insight and self-awareness (Israel et al., 2008). An affirming therapist can be critically important for LGBTQ+ young people who may not have a well-established social support system that is accepting of their LGBTQ+ identity. Indeed, a 2019 national survey found that LGBTQ+ youth who had at least one accepting adult in their lives were 40% less likely to report a suicide attempt in the year prior to being surveyed (The Trevor Project, 2019). Thus, mental health providers who are accepting and trained to adequately respond to LGBTQ+ young adults’ needs could be a key avenue for reducing LGBTQ+ mental health disparities, particularly those related to suicidality.

## State of Current Training

Training for mental health professionals in the US does not currently require specific training in LGBTQ+ health, except for the District of Columbia (LGBTQ Cultural Competency Continuing Education Amendment Act, 2016). Research studies with students and early career professionals in counseling, social work, psychology, and psychiatry generally show that they have positive attitudes toward LGBTQ+ people, but limited training to effectively conduct culturally sensitive care (Ali et al., 2016; Grove, 2009; Logie et al., 2007; McCabe & Rubinson, 2008). To address this need and improve the health and well-being of LGBTQ+ people, mental health providers should receive specific training in LGBTQ+ cultural sensitivity. LGBTQ+ cultural sensitivity can be defined as a set of knowledge, skills, attitudes, and beliefs that enable people to work well with, respond effectively to, and be supportive of LGBTQ+ people.

## Policy Alternatives

Mental health professionals in the US work with separate state regulatory agencies for initial and continued licensure. Minimal licensing requirements are specified outside of receiving a degree from an accredited institution, passing a licensure examination, and completing supervised clinical hours. However, some states specify training hours for specific content, such as in identifying and reporting child abuse.

Integrating LGBTQ+ cultural sensitivity into mental health professional licensing requirements can be an effective means of curbing LGBTQ+ mental health inequities at a structural level while reducing state spending. Research by the Williams Institute at UCLA School of Law has outlined the economic impact of LGBTQ+ discrimination in multiple states; these reports highlight that experiences of LGBTQ+ discrimination can have an economic impact by influencing work productivity, employee turnover, and Medicaid expenditures such as those needed for the treatment of depression (Mallory et al., 2017). The integration of LGBTQ+ cultural sensitivity into professional licensure requirements can be pursued through legislation or rules and regulations.

## Legislation

Passing a new state law for additional licensure requirements casts a wide net that captures any person defined as a mental health professional by the respective state’s Public Health Code. Gathering delegate support for a bill on LGBTQ+ cultural sensitivity may be challenging in states whose constituents do not support LGBTQ+ rights. However, gathering testimonies from mental health professionals and LGBTQ+ community members in support of the proposed legislation may help mitigate these challenges.

There are recent examples of some states that have changed mental health professional licensure criteria. For instance, the Michigan human trafficking licensure requirement was enacted through Senate Bill 597 in 2015 as part of a larger bill package that contained several recommendations included in a 2013 Report on Human Trafficking released by a commission co-chaired by the Michigan Attorney General (Michigan Commission on Human Trafficking, 2013). The bill instituted a one-time requirement for all professions under Article 15 (except those licensed under Part 188) of the Michigan Public Health Code which included those in medicine, dentistry, psychology, counseling, social work, and many others. In a groundbreaking bill, Washington, D.C. also passed the LGBTQ Cultural Competency Continuing Education Act of 2016 which requires that all people in health occupations that require licensure must receive two contact hours of continuing education training per licensing cycle (~ 2 years depending on the profession) in LGBTQ cultural competency for license renewal (LGBTQ Cultural Competency Continuing Education Amendment Act, 2016). Similar to the human trafficking requirement in Michigan, this bill applied to a wide array of professions enumerated under the District of Columbia Health Occupations Revisions Act of 1985 (e.g., nursing, dentistry, medicine, psychology, social work, etc.).

## Rules and Regulations

The rulemaking process involves work at the level of federal or state agencies that institute regulations under granted authority. Rules and regulations are typically amended to comply with new pieces of legislation; however, changes can be made if proposed additions fall under the existing authority of the agency. Thus, LGBTQ+ cultural sensitivity can be added to licensing criteria through a notice of proposed rulemaking put forth by individual state mental health professional licensing bodies who are already issued that power by the state.

Different mental health professions are sometimes governed under different state regulatory agencies which complicate the rulemaking route. In California, the Board of Behavioral Sciences (BBS) is responsible for licensed marriage and family therapists, clinical social workers, professional clinical counselors, and educational psychologists, while the California Board of Psychology handles licensed psychologists and psychological assistants. That means that both the BBS and Board of Psychology would have to pursue the rulemaking process to add LGBTQ+ cultural sensitivity into their respective licensure process since both have limited governance over specific professions.

## Recommendations

Licensing bodies, LGBTQ+ advocates, and mental health professional associations should work to propose legislation or rules and regulations that requires a minimum of two credit hours in LGBTQ+ cultural sensitivity training for initial clinical licensure and license renewal. Initiating this change through legislation rather than rulemaking may be more effective if the bill defines mental health professionals by the state’s public health code; this would require that any state regulatory agency subsumed under the code implement new regulations that meet the same standard requirement defined by the law. Language modeled by the LGBTQ Cultural Competency Continuing Education Act of 2016 can be adapted and used by policymakers and advocates in other parts of the US seeking to make similar changes and should include similar guidance on the content of the trainings (see Appendix A). This act was written such that the new licensure requirements were incorporated into existing systems of training, thus minimizing fiscal impact. Establishing specific training hours in LGBTQ+ cultural sensitivity is, therefore, likely to place minimal burden on providers themselves. Any successful attempts integrating these new requirements would greatly benefit from rigorous implementation evaluations that measure both provider and client outcomes to improve the evidence base that these types of trainings have for working with minoritized populations.

## Conclusion

Increasing LGBTQ+ clients’ access to providers who are sensitized to LGBTQ+ cultural issues could improve their well-being. Mental health providers in the US do not have mandated training requirements in LGBTQ+ health, and many practicing clinicians lack adequate knowledge to work with LGBTQ+ clients in a culturally sensitive and affirming way (Grove, 2009; McCabe & Rubinson, 2008). Addressing this knowledge gap through LGBTQ+ cultural sensitivity training is one tool to help promote positive LGBTQ+ mental health.

## Appendix A

### D.C. Law 21-95. LGBTQ Cultural Competency Continuing Education Amendment Act of 2016

#### AN ACT

*To amend the District of Columbia Health Occupations Revision Act of 1985 to require continuing education for health occupations on the subject of cultural competence and appropriate clinical treatment for individuals who are lesbian, gay, bisexual, transgender, gender nonconforming, queer, or questioning their sexual orientation or gender identity and expression.*

BE IT ENACTED BY THE COUNCIL OF THE DISTRICT OF COLUMBIA, that this act may be cited as the "LGBTQ Cultural Competency Continuing Education Amendment Act of 2016."

**Sec. 2.** Section 510 of the District of Columbia Health Occupations Revision Act of 1985, effective March 25, 1986 (D.C. Law 6-99; D.C. Official Code § 3-1205.10), is amended as follows:

**(a)** Subsection (b) is amended as follows:

**(1)** Paragraph (4)(B)(v) is amended by striking the phrase "prophylaxis treatment." and inserting the phrase "prophylaxis treatment; and" in its place.

**(2)** A new paragraph (5) is added to read as follows:

"**(5)(A)** Except as provided in subsection (b-1)(4) of this section, require that any continuing education requirements for the practice of any health occupation licensed, registered, or certified under this section include 2 credits of instruction on cultural competency or specialized clinical training focusing on patients who identify as lesbian, gay, bisexual, transgender, gender nonconforming, queer, or question their sexual orientation or gender identity and expression ("LGBTQ").”

"**(B)** The instruction required by subparagraph (A) of this paragraph shall, at a minimum, provide information and skills to enable a health professional to care effectively and respectfully for patients who identify as LGBTQ, which may include”:

"**(i)** Specialized clinical training relevant to patients who identify as LGBTQ, including training on how to use cultural information and terminology to establish clinical relationships”;

"**(ii)** Training that improves the understanding and application, in a clinical setting, of relevant data concerning health disparities and risk factors for patients who identify as LGBTQ”;

"**(iii)** Training that outlines the legal obligations associated with treating patients who identify as LGBTQ”;

"**(iv)** Best practices for collecting, storing, using, and keeping confidential, information regarding sexual orientation and gender identity”;

"**(v)** Best practices for training support staff regarding the treatment of patients who identify as LGBTQ and their families”;

"**(vi)** Training that improves the understanding of the intersections between systems of oppression and discrimination and improves the recognition that those who identify as LGBTQ may experience these systems in varying degrees of intensity”; and

"**(vii)** Training that addresses underlying cultural biases aimed at improving the provision of nondiscriminatory care for patients who identify as LGBTQ."

**(b)** Subsection (b-1) is amended as follows:

**(1)** Paragraph (2) is amended by striking the word "and" at the end.

**(2)** Paragraph (3) is amended by striking the phrase "considers appropriate." and inserting the phrase "considers appropriate; and" in its place.

**(3)** A new paragraph (4) is added to read as follows:

"**(4)** Waive by rule the requirement in subsection (b)(5) of this section for”:

"**(A)** Any health occupation licensed, registered, or certified under this section if members of that health occupation do not see patients in a clinical setting”; or

"**(B)** Any licensed health professional who can prove to the satisfaction of the relevant board that he or she did not see patients in a clinical setting in the District during the previous licensing cycle."

**Sec. 3.** Fiscal impact statement.

The Council adopts the fiscal impact statement in the committee report as the fiscal impact statement required by Sect. 4a of the General Legislative Procedures Act of 1975, approved October 16, 2006 (120 Stat. 2038; D.C. Official Code § 1-301.47a).

**Sec. 4.** Effective date.
